# Supplementary material for: Mapping the evolution of fertility support policies in China: A content and instrumental analysis
Source: PLoS One. 2025 Oct 9;20(10):e0332137. doi: 10.1371/journal.pone.0332137 (PMC12510515; doi:10.1371/journal.pone.0332137)
Supplement: S1 Appendix — (ZIP) [file pone.0332137.s001.zip › S1 Appendix. 226 original policy documents/165-中华人民共和国母婴保健法实施办法(FBM-CLI-4-13474).doc]

中华人民共和国母婴保健法实施办法

**发布部门：** [卫生部(已撤销)](https://www.pkulaw.com/law/chl?Aggs.IssueDepartment=60222) [机构沿革](javascript:void(0))

**发文字号：**中华人民共和国卫生部令[第45号]

**发布日期：**1995.08.29

**实施日期：**1995.08.29

**时效性：** [失效](https://www.pkulaw.com/law/chl?Aggs.TimelinessDic=02)

**效力级别：** [部门规章](https://www.pkulaw.com/law/chl?Aggs.EffectivenessDic=XE0301)

**法规类别：** [老少妇幼残保护](https://www.pkulaw.com/law/chl?Aggs.Category=01604) [医疗保健](https://www.pkulaw.com/law/chl?Aggs.Category=09623)

**失效依据：**[卫生部决定废止和宣布失效卫生部令目录](javascript:TMP(41928,0,'chl'))

[2017-2022编注](https://www.pkulaw.com/history/compare/编注-4094.html) [2001-2017编注](https://www.pkulaw.com/history/compare/编注-1177.html) [1995-2001对照](https://www.pkulaw.com/history/compare/对照-1179.html)

中华人民共和国卫生部令
（第４５号）

　　现发布《中华人民共和国母婴保健法实施办法》，请各地遵照执行。
　　　　　　　　　　　　　　　　　　　　　　　　　　　　　部长　陈敏章
　　　　　　　　　　　　　　　　　　　　　　　　　一九九五年八月二十九日
　　　　　　　　　　中华人民共和国母婴保健法实施办法

第一章　总则

　　**第一条**　根据《[中华人民共和国母婴保健法](https://www.pkulaw.com/chl/ea633acd04298a40bdfb.html)》（以下简称[《母婴保健法》](https://www.pkulaw.com/chl/ea633acd04298a40bdfb.html)）制定本办法。

　　**第二条**　母婴保健工作实行以保健为中心、保健和临床相结合，面向群体、面向基层和预防为主的工作方针。

　　**第三条**　各省、自治区、直辖市在编制年度及中长期财政预算、配置卫生资源时，应当优先扶持母婴保健事业，对边远贫困地区给予特殊支持，以保障母亲和婴儿获得医疗保健服务。

　　**第四条**　[《母婴保健法》](https://www.pkulaw.com/chl/ea633acd04298a40bdfb.html)第[三条](https://www.pkulaw.com/chl/ea633acd04298a40bdfb.html#tiao_3)第[一款](https://www.pkulaw.com/chl/ea633acd04298a40bdfb.html#tiao_3_kuan_1)规定的各级人民政府领导母婴保健工作是指：

　　（一）将母婴保健事业纳入本地区国民经济和社会发展计划，制定本地区母婴保健工作发展规划，并为规划目标的实现提供政策保障；

　　（二）组织、协调有关部门在各自职责范围内，配合卫生行政部门做好[《母婴保健法》](https://www.pkulaw.com/chl/ea633acd04298a40bdfb.html)的执法监督管理工作；

　　（三）为本地区医疗保健机构贯彻实施[《母婴保健法》](https://www.pkulaw.com/chl/ea633acd04298a40bdfb.html)提供必要条件，物质帮助以及执法的专项经费。

　　**第五条**　卫生部主管全国母婴保健工作，并对母婴保健工作实施监督管理，其主要职责是：

　　（一）执行[《母婴保健法》](https://www.pkulaw.com/chl/ea633acd04298a40bdfb.html)及本办法；

　　（二）制定[《母婴保健法》](https://www.pkulaw.com/chl/ea633acd04298a40bdfb.html)配套规章及技术规范；

　　（三）按照分级分类指导原则制定全国母婴保健工作发展规划和实施步骤；

　　（四）组织鉴定并推广母婴保健适宜技术；

　　（五）对母婴保健工作进行监督管理。

第二章　技术服务机构与人员

　　**第六条**　[《母婴保健法》](https://www.pkulaw.com/chl/ea633acd04298a40bdfb.html)及本办法规定的医疗保健机构是指依据[《母婴保健法》](https://www.pkulaw.com/chl/ea633acd04298a40bdfb.html)开展母婴保健业务的各级妇幼保健机构以及其它开展母婴保健技术服务的机构。

　　**第七条**　医疗保健机构开展婚前医学检查，必须经设区的市级以上卫生行政部门审批，取得《母婴保健技术服务执业许可证》。

　　**第八条**　医疗保健机构和其它开展母婴保健技术服务的机构开展结扎手术和终止妊娠手术，必须经县级以上卫生行政部门审批，取得《母婴保健技术服务执业许可证》。

　　**第九条**　医疗保健机构开展遗传病诊断和产前诊断，必须经省级卫生行政部门审批，取得《母婴保健技术服务执业许可证》。

　　**第十条**　从事[《母婴保健法》](https://www.pkulaw.com/chl/ea633acd04298a40bdfb.html)规定的婚前医学检查、遗传病诊断、产前诊断、结扎手术和终止妊娠手术的人员，应当参加卫生行政部门组织的[《母婴保健法》](https://www.pkulaw.com/chl/ea633acd04298a40bdfb.html)知识培训和业务培训。凡符合卫生部规定的技术人员标准，经考核并取得卫生行政部门颁发的《母婴保健技术考核合格证书》后方可从事以上技术工作。

　　遗传病诊断和产前诊断人员的考核发证，由省、自治区、直辖市卫生行政部门负责。

　　婚前医学检查人员的考核发证由设区的市级以上地方卫生行政部门负责。

　　结扎手术和终止妊娠手术人员的考核发证，由县级以上地方卫生行政部门负责。

　　**第十一条**　从事家庭接生的人员必须参加卫生行政部门组织的[《母婴保健法》](https://www.pkulaw.com/chl/ea633acd04298a40bdfb.html)知识培训和业务培训。凡符合卫生部规定的技术人员标准和要求并经考核合格及取得县级卫生行政部门颁发的《家庭接生员技术合格证书》后方可从事正常产的家庭接生工作。

　　**第十二条**　未取得《母婴保健技术服务执业许可证》的医疗保健机构和未取得《母婴保健技术考核合格证书》、《家庭接生员技术合格证书》的人员不得从事[《母婴保健法》](https://www.pkulaw.com/chl/ea633acd04298a40bdfb.html)规定的婚前医学检查、遗传病诊断、产前诊断、结扎手术、终止妊娠手术和家庭接生。

　　**第十三条**　《母婴保健技术服务执业许可证》、《母婴保健技术考核合格证书》、《家庭接生员技术合格证书》有效期三年。有效期满后继续开展母婴保健技术服务的，由原发证机关重新审核认可。

第三章　婚前保健

　　**第十四条**　医疗保健机构应当开展婚前健康教育和婚前卫生咨询服务。

　　婚前医学检查由县级以上妇幼保健院或经设区的市级以上卫生行政部门指定的医疗机构承担。

　　**第十五条**　开展婚前医学检查的医疗保健机构应当具备下列条件：

　　（一）分别设置专用的男、女婚前医学检查室，配备常规检查和专科检查设备；

　　（二）设置婚前健康教育宣教室；

　　（三）有合格的专职男、女婚前医学检查医师。

　　**第十六条**　婚前医学检查包括询问病史、体格及相关检查。婚前医学检查项目按照卫生部有关婚前保健工作常规执行。

　　[《母婴保健法》](https://www.pkulaw.com/chl/ea633acd04298a40bdfb.html)第[八条](https://www.pkulaw.com/chl/ea633acd04298a40bdfb.html#tiao_8)规定的严重遗传性疾病、指定传染病和有关精神病的病种，除法律已有规定外，由卫生部另行公布。

　　**第十七条**　准备结婚的男女双方在办理结婚登记前，应当持下列材料到任何一方户籍所在地的婚前医学检查单位进行婚前医学检查：

　　（一）《居民身份证》、户籍证明或其他有效身份证明；

　　（二）近期一寸免冠照片三张。

　　涉外婚姻当事人应当到省级卫生行政部门批准的婚前医学检查单位进行婚前医学检查。

　　**第十八条**　婚前医学检查实行逐级转诊制度。对不能确诊的，应当转到设区的市级以上卫生行政部门指定的医疗保健机构进行确诊。原婚前医学检查单位应当根据确诊结果填写《婚前医学检查证明》。

　　**第十九条**　婚前医学检查单位应当向接受婚前医学检查的当事人出具《婚前医学检查证明》。

　　对患指定传染病在传染期内和有关精神病在发病期内的，医师应当提出医学意见，并如实填写《婚前医学检查证明》。

　　对患医学上认为不宜生育的严重遗传性疾病的，医师应当向当事人说明情况，提出医学意见，并如实填写《婚前医学检查证明》；对已经采取长效避孕措施或施行结扎手术的，应当予以注明。

　　不宜生育的严重遗传性疾病的诊断由省级卫生行政部门指定的医疗保健机构负责。

第四章　孕产期保健

　　**第二十条**　按照当地卫生行政部门划定的服务区域，医疗保健机构应当在各自的职责范围内，为育龄妇女和孕产妇提供生殖健康和孕产期保健服务。

　　**第二十一条**　孕产期保健服务指从怀孕开始至产后四十二天内为孕产妇及胎、婴儿提供的医疗保健服务。其主要工作是：

　　（一）为孕育健康后代提供医学指导与咨询；

　　（二）为孕妇建立孕产妇保健手册（卡），定期为孕妇进行产前检查，记录检查结果；

　　（三）为孕妇提供营养及孕期自我保健指导；

　　（四）针对高危孕妇做好保健管理工作，采取适宜技术对孕产妇及胎儿进行监护，使具有不同危险因素的孕妇能按其危险程度，得到相应的保健医疗服务；

　　（五）提倡住院分娩，做好消毒接生和新生儿复苏工作，采用适宜技术对孕产妇及胎儿进行监护，预防产伤及产后出血，降低孕产妇及围产儿的发病率、死亡率；

　　（六）实行母乳喂养，指导产妇科学哺乳，执行母乳喂养十条标准；

　　（七）定期进行产后访视，有条件的地区开展新生儿疾病筛查工作；

　　（八）为贫困地区或新生儿破伤风高发地区育龄妇女，特别是孕妇进行破伤风类毒素预防接种；

　　（九）提供避孕措施方面的咨询指导和技术服务。

　　（十）对产妇和家属开展健康教育和科学育儿知识教育。

　　**第二十二条**　[《母婴保健法》](https://www.pkulaw.com/chl/ea633acd04298a40bdfb.html)第[十五条](https://www.pkulaw.com/chl/ea633acd04298a40bdfb.html#tiao_15)规定的严重疾病是指：

　　（一）妊娠合并严重的心、肝、肺、肾疾病和糖尿病；

　　（二）严重精神性疾病；

　　（三）严重的妊娠高血压综合症；

　　（四）省级以上卫生行政部门规定的严重影响生育的其它疾病。

　　**第二十三条**　[《母婴保健法》](https://www.pkulaw.com/chl/ea633acd04298a40bdfb.html)第[十五条](https://www.pkulaw.com/chl/ea633acd04298a40bdfb.html#tiao_15)规定的致畸物质是指可能导致胎儿发生先天缺陷的有害物质，包括化学（如苯、有机汞、农药、某些药物等）、生物（如病毒、弓形体等）、物理（如放射线、同位素等），以及省级以上卫生行政部门规定的其它化学、生物、物理的致畸性有害物质。

　　**第二十四条**　女职工保健按照卫生部、劳动部、人事部、全国总工会、全国妇联五个部门联合颁发的《[女职工保健工作规定](https://www.pkulaw.com/chl/6bd61140293005a1bdfb.html)》执行。

　　**第二十五条**　发现孕妇有下列情形之一的，应当进行产前诊断：

　　（一）羊水过多或过少；

　　（二）胎儿发育异常或胎儿可能有畸形；

　　（三）孕早期接触过可能导致胎儿先天缺陷的物质；

　　（四）曾经分娩过先天性严重缺陷的婴儿；

　　（五）年龄超过３５周岁；

　　（六）省级以上卫生行政部门规定的其它情形。

　　**第二十六条**　[《母婴保健法》](https://www.pkulaw.com/chl/ea633acd04298a40bdfb.html)第[十六条](https://www.pkulaw.com/chl/ea633acd04298a40bdfb.html#tiao_16)、第[十八条](https://www.pkulaw.com/chl/ea633acd04298a40bdfb.html#tiao_18)第[一项](https://www.pkulaw.com/chl/ea633acd04298a40bdfb.html#tiao_18_kuan_1_xiang_1)规定的严重遗传性疾病的种类由卫生部另行公布。

　　**第二十七条**　[《母婴保健法》](https://www.pkulaw.com/chl/ea633acd04298a40bdfb.html)第[十八条](https://www.pkulaw.com/chl/ea633acd04298a40bdfb.html#tiao_18)第[二项](https://www.pkulaw.com/chl/ea633acd04298a40bdfb.html#tiao_18_kuan_1_xiang_2)规定的严重缺陷是指：

　　（一）无脑畸形、脑积水、脊柱裂、脑脊膜膨出等；

　　（二）内脏膨出或内脏外翻；

　　（三）四肢短小畸形；

　　（四）其它严重的胎儿畸形。

　　**第二十八条**　依照[《母婴保健法》](https://www.pkulaw.com/chl/ea633acd04298a40bdfb.html)规定施行终止妊娠或者结扎手术的费用；按劳保医疗、公费医疗的规定予以报销；手术者不享受劳保医疗、公费医疗的，应当由地方政府设立的专项经费解决。

　　**第二十九条**　生育过严重遗传性疾病或严重缺陷患儿的，再次妊娠前，夫妻双方应当到县级以上妇幼保健院或省级卫生行政部门指定的医疗机构进行医学检查。对确诊患有医学上认为不宜生育的严重遗传性疾病的，医师应当向当事人说明情况，并提出医学意见。

　　**第三十条**　提倡住院分娩。高危孕妇必须在有条件的医疗保健机构住院分娩。

　　没有条件住院分娩的正常产，应当由取得《家庭接生员技术合格证书》的人员接生。

　　**第三十一条**　医疗保健机构根据接生人员签署的出生医学记录出具《出生医学证明》。

　　《出生医学证明》应当加盖接生单位的“出生医学证明专用章”。

　　《出生医学证明》是新生儿申报户口的依据。

　　家庭接生的，由所在乡（镇）卫生院出具《出生医学证明》。

　　**第三十二条**　医疗保健机构应当加强对母婴保健人员的岗位业务培训和职业道德教育，定期检查、考核其业务水平和职业道德。

　　医师和助产人员（包括家庭接生人员）应当严格遵守有关操作常规，认真填写各项记录，提高助产技术和服务质量。

　　助产人员管理办法由卫生部另行制定。

　　**第三十三条**　严禁采用技术手段对胎儿进行性别鉴定。但怀疑胎儿为伴性遗传病，严重Ｘ连锁智力低下的，经医疗保健机构领导批准可以做性别鉴定。

　　**第三十四条**　国家建立孕产妇死亡、婴儿死亡和新生儿出生缺陷报告制度。其管理办法由卫生部制定。

第五章　婴儿保健

　　**第三十五条**　母乳喂养是婴儿的权利，全社会都要保护和支持母乳喂养，在全国范围内严格执行《[母乳代用品销售管理办法](https://www.pkulaw.com/chl/f4e95820217863adbdfb.html)》。医疗保健机构应当为母乳喂养提供必要条件。

　　**第三十六条**　医疗保健机构应当对新生儿进行登记，建立儿童保健手册，并对新生儿进行访视，对婴幼儿进行定期健康检查和预防接种。

　　**第三十七条**　医疗保健人员进行新生儿访视和婴儿健康检查时，应当提供有关母乳喂养、合理膳食、健康心理行为等科学育儿知识。

　　**第三十八条**　医疗保健机构应当开展新生儿先天性甲状腺功能低下和苯丙酮尿症等疾病的筛查，并提出治疗意见。

　　**第三十九条**　医疗保健机构应当开展婴幼儿常见病、多发病的防治工作，并提供眼、耳、口腔保健及促进婴儿神经、精神发育的有关服务。对高危、体弱者应当重点监护。

　　**第四十条**　医疗保健机构应当按照国家教委和卫生部颁发的《[托儿所、幼儿园卫生保健管理办法](https://www.pkulaw.com/chl/2b1ca11657cd7b4abdfb.html)》对托幼园、所卫生保健工作实行统一管理和监督。

第六章　技术鉴定

　　**第四十一条**　县级以上地方人民政府设立的母婴保健医学技术鉴定组织，称为母婴保健医学技术鉴定委员会。

　　母婴保健医学技术鉴定委员会成员由卫生行政部门提出人选，报同级人民政府聘任。

　　**第四十二条**　母婴保健医学技术鉴定委员会成员应当由医德高尚、具有丰富医疗保健实践经验和相关学科理论知识，具备主治医师以上的专业技术职务的专业人员组成。

　　**第四十三条**　母婴保健医学技术鉴定委员会负责本行政区域内有异议的婚前医学检查、遗传病诊断、产前诊断的结果和有异议的下一级医学技术鉴定结论的医学技术鉴定工作。

　　**第四十四条**　母婴保健医学技术鉴定分为省、市、县三级鉴定。省级母婴保健医学技术鉴定委员会的鉴定为最终鉴定结论。

　　**第四十五条**　对婚前医学检查、遗传病诊断、产前诊断的结果有异议的，可以在接到结果之日起１５日内向所在地母婴保健医学技术鉴定委员会申请医学技术鉴定。

　　**第四十六条**　要求进行医学技术鉴定的，应当向母婴保健医学技术鉴定委员会提出书面申请，填写《母婴保健技术鉴定申请表》，并提交有关材料。

　　母婴保健医学技术鉴定委员会应当在接到鉴定申请之日起３０日内作出医学技术鉴定结论，如有特殊情况，一般不得超过９０日，并及时通知当事人。

　　当事人对鉴定结论有异议可在接到鉴定结论通知书之日起１５日内向上一级母婴保健医学技术鉴定委员会申请重新鉴定。

　　**第四十七条**　母婴保健医学技术鉴定委员会进行医学技术鉴定时必须有五名以上相关专业鉴定委员会成员参加。与申请鉴定的当事人有利害关系的人员应当回避。

　　参加鉴定的鉴定委员会成员应当在鉴定结论上签名，对不同意见应当如实记录。与鉴定有关的材料和鉴定结论原件必须立卷存档。

　　**第四十八条**　申请医学技术鉴定的鉴定费用由申请人预付，根据鉴定结论，由责任人支付。

第七章　管理与监督

　　**第四十九条**　各级卫生行政部门要设立母婴保健执法监督的机构或配备政治素质好，能承担执法监督任务的人员，负责母婴保健执法监督工作。

　　**第五十条**　各级卫生行政部门负责妇幼保健机构的规划设置、执业登记、注册、校验和有关监督工作。

　　政府要扶持贫困地区妇幼保健机构，使其达到妇幼保健院基本标准，能肩负起依法提供母婴保健服务的任务。

　　**第五十一条**　妇幼保健机构的设置、服务范围按照卫生部有关妇幼保健机构分级分等标准和妇幼卫生服务规范执行。

　　**第五十二条**　县级以上地方人民政府卫生行政部门对本行政区域内的母婴保健工作实施监督管理，其主要职责是：

　　（一）按照卫生部规定的条件和技术标准，对申请从事婚前医学检查、遗传病诊断、产前诊断以及结扎手术和终止妊娠手术的医疗保健机构进行审批；

　　（二）按照卫生部规定的条件和技术标准对从事婚前医学检查、遗传病诊断、产前诊断、结扎手术和终止妊娠手术的人员以及从事家庭接生的人员进行考核，并颁发相应的证书；

　　（三）对[《母婴保健法》](https://www.pkulaw.com/chl/ea633acd04298a40bdfb.html)对本办法的执行情况进行监督检查；

　　（四）依照[《母婴保健法》](https://www.pkulaw.com/chl/ea633acd04298a40bdfb.html)和本办法决定行政处罚。

　　**第五十三条**　县级以上卫生行政部门设立母婴保健监督员。母婴保健监督员主要从卫生行政部门聘任，根据需要也可以从妇幼保健院中选聘有一定管理经验和技术水平的人员担任。

　　母婴保健监督员由同级卫生行政部门审核发证，报上一级卫生行政部门备案。

　　**第五十四条**　母婴保健监督员的主要职责是：

　　（一）监督检查[《母婴保健法》](https://www.pkulaw.com/chl/ea633acd04298a40bdfb.html)与本办法的执行情况；

　　（二）对违反[《母婴保健法》](https://www.pkulaw.com/chl/ea633acd04298a40bdfb.html)与本办法的单位和个人提出处罚意见；

　　（三）对母婴保健工作提出改进建议；

　　（四）完成卫生行政部门交给的其它监督管理任务。

　　**第五十五条**　母婴保健监督员的管理依照卫生部《[母婴保健监督员管理办法](https://www.pkulaw.com/chl/04d4343537ed0d3bbdfb.html)》执行。

　　**第五十六条**　《婚前医学检查证明》的格式由卫生部规定，各省、自治区、直辖市印制。

　　《出生医学证明》、《母婴保健技术服务执业许可证》、《母婴保健技术考核合格证书》、《家庭接生员技术合格证书》、《母婴保健监督员证》由卫生部统一印制。

第八章　处罚

　　**第五十七条**　未取得《母婴保健技术服务执业许可证》、《母婴保健技术考核合格证书》和《家庭接生员技术合格证书》，而从事婚前医学检查、遗传病诊断、产前诊断、结扎手术和终止妊娠手术和家庭接生以及出具[《母婴保健法》](https://www.pkulaw.com/chl/ea633acd04298a40bdfb.html)规定的婚前医学检查证明、新生儿出生医学证明和医学技术鉴定证明的，由县级以上地方人民政府卫生行政部门予以制止，并给予以下处罚：

　　（一）责令停止；

　　（二）警告；

　　（三）处以５００元以上５０００元以下的罚款；情节严重或经制止仍不改正的，处以５０００元以上２００００元以下的罚款。

　　以上处罚可单独或合并使用。

　　**第五十八条**　从事母婴保健专项技术服务工作和医学技术鉴定的人员出具虚假医学证明文件或违反[《母婴保健法》](https://www.pkulaw.com/chl/ea633acd04298a40bdfb.html)规定进行胎儿性别鉴定的，由所在医疗保健机构或卫生行政部门根据情节给予行政处分；有下列情形之一的，由卫生行政部门取消其执业资格：

　　（一）出具虚假医学证明或违法进行胎儿性别鉴定经制止仍不改正的；

　　（二）出具虚假医学证明给当事人造成严重后果的；

　　（三）违法进行胎儿性别鉴定给当事人身心造成严重伤害的。

第九章　附则

　　**第五十九条**　本办法的解释权在卫生部。

　　**第六十条**　本办法自发布之日起施行。

© 北大法宝：（[www.pkulaw.com](https://www.pkulaw.com/)) 专业提供法律信息、法学知识和法律软件领域各类解决方案。北大法宝为您提供丰富的参考资料，正式引用法规条文时请与标准文本核对。
欢迎查看所有[产品和服务](https://www.pkulaw.com/helps/2.html)。法宝快讯：[如何快速找到您需要的检索结果？](https://www.pkulaw.com/helps/70.html)[法宝](https://www.pkulaw.com/helps/69.html)V6有何新特色？

扫描二维码阅读原文

**原文链接：**<https://www.pkulaw.com/chl/e833957e60aa0514bdfb.html>
